# Setting the Research Agenda for Clinical Artificial Intelligence in Pancreatic Adenocarcinoma Imaging

**DOI:** 10.3390/cancers14143498

**Published:** 2022-07-19

**Authors:** Megan Schuurmans, Natália Alves, Pierpaolo Vendittelli, Henkjan Huisman, John Hermans

**Affiliations:** 1 Diagnostic Image Analysis Group, Radboud University Medical Center, 6500 HB Nijmegen, The Netherlands; pierpaolo.vendittelli@radboudumc.nl (P.V.); henkjan.huisman@radboudumc.nl (H.H.); 2 Department of Medical Imaging, Radboud University Medical Center, 6500 HB Nijmegen, The Netherlands; john.hermans@radboudumc.nl

**Keywords:** pancreatic cancer, artificial intelligence, imaging, radiology, pathology

## Abstract

**Simple Summary:**

Pancreatic ductal adenocarcinoma (PDAC) is one of the deadliest cancers worldwide, associated with a 98% loss of life expectancy and a 30% increase in disability-adjusted life years. Image-based artificial intelligence (AI) can help improve outcomes for PDAC given that current clinical guidelines are non-uniform and lack evidence-based consensus. However, research on image-based AI for PDAC is too scattered and lacking in sufficient quality to be incorporated into clinical workflows. In this review, an international, multi-disciplinary team of the world’s leading experts in pancreatic cancer breaks down the patient pathway and pinpoints the current clinical touchpoints in each stage. The available PDAC imaging AI literature addressing each pathway stage is then rigorously analyzed, and current performance and pitfalls are identified in a comprehensive overview. Finally, the future research agenda for clinically relevant, image-driven AI in PDAC is proposed.

**Abstract:**

Pancreatic ductal adenocarcinoma (PDAC), estimated to become the second leading cause of cancer deaths in western societies by 2030, was flagged as a neglected cancer by the European Commission and the United States Congress. Due to lack of investment in research and development, combined with a complex and aggressive tumour biology, PDAC overall survival has not significantly improved the past decades. Cross-sectional imaging and histopathology play a crucial role throughout the patient pathway. However, current clinical guidelines for diagnostic workup, patient stratification, treatment response assessment, and follow-up are non-uniform and lack evidence-based consensus. Artificial Intelligence (AI) can leverage multimodal data to improve patient outcomes, but PDAC AI research is too scattered and lacking in quality to be incorporated into clinical workflows. This review describes the patient pathway and derives touchpoints for image-based AI research in collaboration with a multi-disciplinary, multi-institutional expert panel. The literature exploring AI to address these touchpoints is thoroughly retrieved and analysed to identify the existing trends and knowledge gaps. The results show absence of multi-institutional, well-curated datasets, an essential building block for robust AI applications. Furthermore, most research is unimodal, does not use state-of-the-art AI techniques, and lacks reliable ground truth. Based on this, the future research agenda for clinically relevant, image-driven AI in PDAC is proposed.

## 1. Introduction

Pancreatic cancer is one of the deadliest cancers worldwide, with a 5-year survival rate of less than 5% [1]. Pancreatic ductal adenocarcinoma (PDAC), the most common and aggressive type of pancreatic cancer, has become a medical emergency in the past decades. PDAC tumours present highly aggressive behavior, leading to 98% life expectancy loss and a 30% increase in disability-adjusted life years [2,3]. Recently, there have been some improvements in the survival rates of early-stage, resected patients following neo-adjuvant chemotherapy [4]. However, this translates into negligible increase in survival for the whole PDAC patient population as in the absence of effective prevention and screening protocols, most patients are diagnosed with advanced disease [4]. Still, research funding for PDAC remains significantly lower than for other cancer types, leading it to be flagged as a neglected cancer by both the European Commission and the United States Congress [2].

Cross-sectional imaging, namely computed tomography (CT), magnetic resonance (MR), 18fluoro-2-deoxy-d-glucose positron emission tomography/computed tomography (18FDG PET/CT), and endoscopic ultrasound (EUS), play a crucial role in PDAC management. Nevertheless, current international guidelines for image-based stratification, treatment response prediction, and evaluation are heterogeneous and ineffective [5]. Histopathology analysis is considered the gold standard for PDAC diagnosis and characterization. Still, it remains challenging even for experienced pathologists due to marked morphological tumour heterogeneity and the limited amount of tumour tissue in biopsy [6,7,8]. Moreover, histopathology evaluation of treatment response is imprecise, of limited clinical relevance, and affected by interobserver variation [9].

Artificial intelligence (AI) has gained considerable interest in oncology, as it has the potential to leverage high amounts of data to produce individualised recommendations based on each patient’s clinical picture [10]. As the volume of multi-modal data acquired in routine clinical practice increases, AI can support clinicians and ultimately guide decision making at each step of the patient pathway by focusing on well-validated applications at meaningful clinical touchpoints [10]. Commercial clinical AI is already a reality for diseases such breast, colon, and lung cancer, with multiple FDA-approved products on the market for applications such as disease screening, diagnosis, and tumour characterization [11]. Currently, there are two main approaches for image-based AI: radiomics and convolutional neural networks (CNNs). Radiomics predicts an outcome by feeding manually defined texture and shape features extracted from a region of interest to machine learning models. Deep learning-based CNNs, on the other hand, automatically compute the relevant features directly from the imaging during training, in a neural network comprising a sequence of convolutional and pooling operations. Since the introduction of AlexNet in 2012, CNNs have evolved enormously and are now dominating image analysis, but the transition from hand-crafted radiomic features to deep learning in the medical domain has been gradual [12,13].

The number of publications on AI for clinical decision-making in oncology has increased exponentially in the past few years [12]. However, AI research in PDAC is still at a preliminary stage compared to other cancer diseases, with limited private and public datasets and a lack of independent, external model validation. As a result, no AI applications have been implemented in clinical practice for PDAC.

The contributions of this review are threefold. First, an international, multi-disciplinary, multi-institutional expert panel breaks down the PDAC patient clinical pathway and identifies the most relevant clinical questions for image-based AI research. Second, we analyse the available literature addressing these clinical questions and identify current trends and knowledge gaps. Finally, we define the research agenda for clinical AI research in PDAC imaging, along with the necessary steps towards clinically relevant AI applications that can improve patient outcomes.

## 2. PDAC Patient Pathway

**Figure 1:**
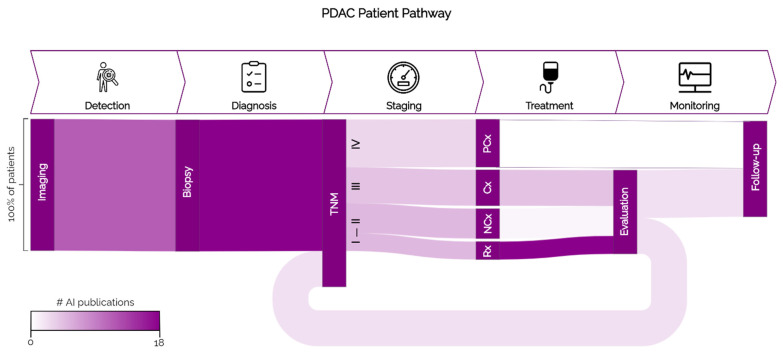
PDAC patient pathway. The steps of the general cancer patient pathway are shown in the top part of the figure. Below, the vertical boxes show the actions/guidelines for PDAC used in each step. The width of the streams represents the proportion of patients that go through each branch of the pathway, and the colours of the streams represent the number of AI publications found on that topic. Rx: resection; nCTx: neoadjuvant chemo(radio)therapy; aCTx: adjuvant/induction therapy; Px: palliative care.

The typical cancer patient pathway is generally subdivided into five steps: detection, diagnosis, staging, treatment, and monitoring. In each step of the patient pathway there are critical patient and clinician decision-oriented touchpoints that could benefit from AI [10]. These touchpoints are specific for each cancer subtype and regard clinical decisions that are suboptimal with currently implemented workflows and guidelines [10]. The specific steps of the PDAC patient pathway are illustrated in Figure 1, and the clinical touchpoints for potential AI development in each step are explored in the following sections.

### 2.1. Detection

Timely detection is crucial to improve PDAC patients’ outcomes, as the 5-year survival increases from only 3% in metastatic patients to 42% when the tumour is still confined to the primary site [14]. According to the Japan Pancreatic Cancer Registry, patients in the earliest disease stage show a survival rate as high as 80.4% but account for only 0.8% of cases [15]. Due to the low incidence of PDAC, defining and screening groups at risk is a vital step to improve patient outcome. Research on risk factors, new screening protocols, and non-invasive tumour biomarkers is on the rise, but so far there are no validated biomarkers or tools for early detection. Therefore, screening is still not part of the PDAC patient pathway as it is cost-prohibitive with current technology. The most used modality for PDAC detection is multi-phase contrast-enhanced CT (CECT). However, early PDAC detection on CECT remains challenging, as lesions are small (size <2 cm), present poorly defined margins, and are more often iso-attenuating [5,16]. Radiologists’ sensitivity at detecting lesions with size smaller than 2 cm on CECT has been reported to be as low as 58% [5,16]. Contrast-enhanced MRI is highly effective at detecting tumours that are poorly visible on CECT, but is not yet routinely implemented in the clinic [17]. EUS is a widely accepted modality for the diagnosis of PDAC.

Early detection can be facilitated by the timely identification of secondary imaging signs predictive of PDAC, such as main pancreatic duct cut-off or dilation, parenchymal atrophy, and irregular pancreatic contour [5,18]. These signs are often visible on CECT scans 18 to 12 months prior to clinical diagnosis, but the reported radiologists’ sensitivity for their timely detection is only 44%, limiting the chances of early action [18].

### 2.2. Diagnosis

PDAC symptoms are mostly unspecific in early disease stages, and as lesional appearances are heterogeneous on CECT, patients are often initially misdiagnosed with other, more common abdominal diseases with similar symptomatology (e.g., gallbladder diseases, acute or chronic pancreatitis, duodenum cancer) [18,19]. Initially misdiagnosed patients are reported to present higher rates of abdominal pain, weight loss, and acute pancreatitis than correctly diagnosed patients, and are at a higher risk of advanced disease [19]. EUS is also a widely accepted imaging modality for the diagnosis of PDAC, especially for lesions less than 2–3 cm in size in which it reaches superior sensitivity compared to CT [20]. Furthermore, EUS has a high negative predictive value and can be used to reliably exclude pancreatic cancer [20]. Histopathology assessment is the current gold standard for PDAC diagnosis confirmation and is usually based on EUS fine-needle cytology or biopsy. Nevertheless, the morphological distinction of PDAC from other lesions on small biopsies or cytology samples can be challenging, especially given the minimal amount of lesional material that is often contained in these samples [21].

### 2.3. Staging

Following histopathology diagnosis, the most used method for PDAC staging is the TNM classification by the American Joint Committee on Cancer (AJCC). The local tumour extent (T stage), the dissemination to the regional lymph nodes (N stage), and the metastatic spread to distant sites (M stage) are used to stratify patients, determine their prognosis, and indicate treatment and monitoring strategy [19]. Nevertheless, the TNM classification’s predictiveness for overall survival (OS) is not reliable [21]. A 2018 multicentre study aiming to validate the AJCC TNM 8th edition in a cohort of 1525 patients receiving pancreatoduodenectomy reported a concordance index of 0.57 (95% CI, 0.55–0.60) for OS prediction [22].

### 2.4. Treatment

The most common treatment options for PDAC are resection and chemo(radio)therapy, in particular using FOLFIRINOX and gemcitabine–abraxane [2]. Surgical resection (Rx) is the only option for potential long-term survival, but as can be seen in Figure 1 is only suitable for a minority (10–15%) of patients (stages I, II). Most patients are diagnosed in later disease stages (III, IV) where Rx is no longer possible due to metastasis or extensive vessel involvement [23]. Imaging assessment of tumour-vascular contact primarily determines eligibility for Rx, but there are no widely accepted, evidence-based guidelines for the appropriate tumour resectability criteria [5,24]. As a result, the 5-year survival rate of resected PDAC patients is only 30–58%, with 69–75% of patients relapsing within two years [1,25].

As illustrated in Figure 1, most patients receive chemo(radio)therapy at some point during treatment [4]. Neo-adjuvant chemo(radio)therapy (nCTx) intends to optimise surgical outcome in patients with resectable disease, while adjuvant chemo(radio)therapy (aCTx) is used to downstage unresectable patients. After aCTx, patients may become resectable and undergo Rx or be referred to palliative care (Px), which is intended to suppress disease-related pain and lengthen the patient’s life. Although most patients experience chemotherapy-induced toxicity, often with limited efficacy due to biological resistance, a priori prediction of chemotherapy response is still not possible in current clinical work-up [26,27].

### 2.5. Treatment Monitoring

Following curative resection, histopathology analysis of the resected specimen is performed to confirm the diagnosis of PDAC and to map the extent of disease. This includes the assessment of lymph node metastases (LNM), tumour permeation along lymphatics/blood vessels, and the clearance to the resection margins (resection margin status) [28]. Nevertheless, the prognostic value of these parameters is still controversial, with several studies reporting no significant relationship to survival [28,29,30]. The main reasons for the low predictive power of histopathology findings are the lack of standardised evaluation, consensus definitions, and reporting approaches [31,32].

In patients undergoing chemo(radio)therapy, imaging is critical for determining therapeutic response and selection of the next treatment approach, as acquiring a biopsy could lead to an increase of inflammation [32]. The Response Evaluation Criteria in Solid Tumours (RECIST) 1.1 (2009) is the current standard to evaluate chemo(radio)therapy. This is a purely morphological criteria that quantitatively tracks tumour burden changes based on alterations to the lesions’ size. Although RECIST shows some success in monitoring response based on metastases assessment, it is ineffective when considering the primary tumour, as PDAC lesions present poorly defined borders and significant heterogeneity in regression/progression patterns [32]. Furthermore, chemo(radio)therapy often results in necrotic, fibrous, or inflammatory changes, which translate into an apparent enlargement of the lesion in CT/MRI scans that can be misinterpreted as tumour progression [32].

Current histopathological tumour regression grade (TRG) systems for PDAC are based on a semiquantitative evaluation of the destruction of cancer cells, the amount of residual viable cancer, or the extent of fibrosis induced by treatment. However, current TRG systems are based on imprecise, difficult-to-apply criteria, and a standardised and widely accepted grading system for the histological evaluation of TRG in pancreatic cancer has not yet been established [9,33,34]. These factors make RECIST and histopathology TRG insufficient for predicting local oncological response in PDAC patients [31,32].

## 3. Materials and Methods

Searches were conducted on PubMed, Web of Science, Cochrane, and Embase on 14 September 2021 and updated on 25 January 2022. Additional information about the search strategy can be found in Appendix A and Table A1. Articles were included for evaluation if patient information was available, cohort size was larger than 20 patients, AI was developed to predict a given outcome related to PDAC, and the proposed AI model used imaging (CT, MRI, EUS, PET-CT, whole-slide images (WSI)) as input. Articles were excluded if the research used non-human subjects, did not show any performance, did not report how results were validated.,or used the same cohort for training and reporting of the results.

## 4. Results

**Figure 2:**
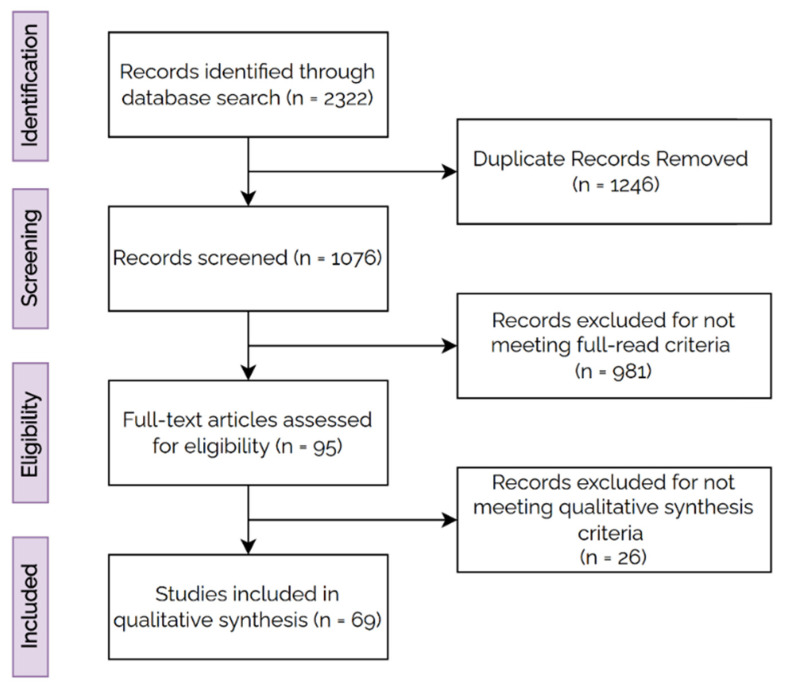
PRISMA Flowchart for inclusion criteria.

A total of 2322 records were retrieved from the electronic databases, and 1076 articles remained after duplicate removal. Titles and abstracts were reviewed on the basis of the inclusion criteria, and 95 articles were eligible for full-text screening. Finally, a total of 69 studies fulfilled the inclusion criteria and were considered for analysis. The flowchart for the inclusion of studies is shown in Figure 2.

### 4.1. Detection

Eleven articles addressed AI for automated PDAC detection (Table 1). Only three articles stratified the results based on tumour size, reporting model performance for the subgroup of lesions with sizes smaller than 2 cm [35,36,37]. Two papers (Alves et al. (2022) and Wang et al. (2021)) reported the results for both lesion detection and localization, and only one paper proposed a fully automatic approach (Alves et al. (2022)) [35,38]. The study by Liu et al. (2020) was the only one comparing AI performance to radiologists based on the analysis of radiology reports, but no reader study was conducted [37]. As is shown in Table 1, only three studies externally tested the proposed models, and four articles used internal cross-validation without separate testing set [35,36,37,38,39,40,41].

### 4.2. Diagnosis

Eighteen papers explored AI for differential PDAC diagnosis (Table 2). The majority of papers (14/18) focused on radiology imaging, mostly (13/14) regarding binary classification between PDAC and another type of lesion, with only one paper tackling multiclass classification [46]. Three publications focused on AI for the histopathological diagnosis of PDAC. Fu et al. (2021) and Naito et al. (2021) proposed DL approaches for PDAC diagnosis and segmentation in WSI, while Kriegsmann et al. (2021) were the first to utilise DL to automatically identify different anatomical tissue structures and diseases on WSI [8,47,48]. AI validation is limited. Only three studies externally tested the proposed models, while nine papers had internal cross-validation, without a separate testing set [8,46,49].

### 4.3. Staging

Thirteen AI papers covered staging (Table 3). Only one publication considered histopathological data. Two articles (An et al., (2021) and Chaddad et al., (2020)) used DL, with the remaining majority using radiomics [63,64]. Most papers considered surrogate end points (histological grade of differentiation, presence of LNM, etc.) as ground truth for model development, with only one considering OS. The study from Chaddad et al., (2021) divided patients into short- and long-term survivors with a set threshold [64]. Only two papers used an external dataset to validate their performance [65,66].

### 4.4. Treatment

Twenty-two studies use pre-treatment imaging to predict treatment response, with the majority of studies (17/22) focusing on patients diagnosed with resectable disease (Table 4). Eleven studies expressed treatment response by predicting OS, of which two (Healy et al., and Zhang et al.) validated the performance in an external cohort [79,80]. Six articles used deep learning (three with the same cohort), with the remaining 16 using radiomics.

### 4.5. Treatment Monitoring

We found two publications regarding treatment evaluation and no publications for follow-up. The study by Janssen et al. (2021) takes a step in the direction of more objective and reproducible TRG systems for patients undergoing nCTx by automatically segmenting relevant structures on WSI of resection specimens [100]. The authors used a cohort of 64 specimens and achieved F1-scores of 0.86 ± 0.09, 0.74 ± 0.12, and 0.86 ± 0.07 for the segmentation of tumour, normal ducts, and remaining non-tumour epithelium, respectively. Nasief et al. (2019) proposed an AI model based on delta radiomics from daily longitudinal scans to predict response to neoadjuvant chemoradiation therapy [101]. This study included 90 patients, divided into good and poor responders based on a modified Ryan Scheme for histopathology-based TRG, and the model achieved an AUC of 0.98 in the independent test set (40 patients) [101].

## 5. Discussion

Clinically relevant AI is developed to assist, replace, or go beyond clinicians’ knowledge on solving problems that affect patient outcomes. AI can significantly impact healthcare by leveraging big data, especially in neglected diseases such as PDAC, but it is essential that research is performed at high-quality standards and focuses on clinical validity, utility, and usability [10].

There are critical steps in the PDAC patient pathway where clinical guidelines are still lacking. In this review, such moments and subsequent opportunities for AI research are identified in consensus by a consortium of radiologists, pathologists, and AI experts from multiple international institutions. We propose that for radiology and pathology AI to advance PDAC care, future research should focus on early diagnosis, data-driven tumour characterisation, survival-based patient staging, treatment response prediction, and monitoring.

Early detection, arguably the most pressing issue in PDAC management, is closely linked to identifying small lesions and secondary anatomical signs [102]. However, our results show this is still not considered in AI-based detection research, as there are no studies on pre-diagnostic detection of secondary signs, and most studies do not disaggregate performance based on tumour size/stage. Additionally, there is a lack of research on lesion localization and a general absence of well-curated datasets, with positive and negative cases being retrieved from completely different populations, which does not reflect the clinical landscape and can introduce bias. For AI to improve PDAC detection, it is crucial to acquire and make publicly available well-curated, multimodal datasets that contain a significant proportion of small (<2 cm or even <1 cm) tumours, which should be treated as a subgroup of interest when reporting model performance.

Current research separates detection, which is defined as distinction between PDAC patients and healthy controls, from differential diagnosis, defined as distinction between PDAC and other types of pancreatic lesions. Only one study developed AI for simultaneous detection and characterisation of pancreatic lesions on CECT [46]. The remaining publications focused on binary distinction between PDAC and one other malignancy, limiting the proposed models’ clinical use. Furthermore, it is important to consider that PDAC diagnosis currently relies on high-quality, adequate imaging with multi-phasic scanning protocols, which may not be widely available due to resource limitations. In the future, research should strive towards a single-use case for radiology-based AI in PDAC diagnosis that includes both the detection of a lesion and its correct classification among a variety of pancreatic diseases in accessible, standard-of-care imaging. The current priority is the curation of large datasets with representative percentages of each lesion type and the integration of different imaging modalities that offer complementary information regarding lesion characterisation.

Research in AI for histopathological PDAC diagnosis is scarce. Only three publications were found to address this topic. While histopathology is considered the gold standard for confirming PDAC diagnosis, it is a time-consuming process that suffers from non-uniform implementation in clinical practice and interobserver variability. Developing powerful AI models for histopathological PDAC diagnosis is fundamental to advance AI research at all steps of the patient pathway. Such models would optimise clinical workflows and empower the generation of reliable ground truth, which could be employed to develop AI with other (non-invasive) modalities, in a timely and cost-effective manner.

AI for PDAC staging lacks a solid reference standard. TNM staging and histopathological grade do not correlate sufficiently with OS and suffer from inter-reader variability. Yet, most AI publications (12/13) focused on grade differentiation and LNM prediction. Only one study considered OS as the outcome, dividing patients into short- and long-term survival based on a threshold derived from the development cohort [64]. In the absence of an international consensus that relates surrogate endpoints to survival, AI research using clinically obtained low- and high-grade differentiation and predicting LNM is not clinically relevant. Future AI research should focus on discovering new data-driven staging biomarkers that relate histopathology and imaging to OS.

AI research for treatment response prediction disproportionally focuses on post-surgery patient outcome, with most included papers considering only resectable patients. Given that 80–85% of patients are diagnosed with unresectable disease, AI research on prediction of response to resection will have a minor impact on improving overall PDAC patients’ outcomes [103]. Instead, research efforts should focus on later disease stages, predicting response to (neo-)adjuvant/palliative chemo(radio)therapy. While most papers addressing treatment response considered survival as the outcome measure, there were also publications that aimed at predicting resection margin status and histopathology-based treatment effect. AI should not focus on these endpoints as they do not accurately reflect whether a patient responds to treatment. There were no publications considering multiple treatment options, with all studies focusing on the prediction of response to a single treatment regime. Future AI research should consider multiple treatment options for a given patient, providing the most favourable suggestion based on survival as the outcome measure.

AI research for treatment monitoring is lagging behind, as to date only two publications considered post-treatment imaging to evaluate response. The results from a segmentation network approach (Janssen et al. (2021)) are promising, but they were not validated externally, and further research is necessary to integrate this AI tool into a reliable TRS system for neoadjuvant chemotherapy [100]. Nasief et al. (2019) used longitudinal scans to monitor chemoradiotherapy response, but the authors considered the histopathology-based treatment response as ground truth [101]. Clinically relevant AI applications should directly predict OS and recurrence from large, well-curated radiology and pathology datasets. Additionally, AI algorithms for treatment monitoring should strive to assist clinicians by indicating the best action at a given time-point, such as timely termination of treatment to prevent unnecessary comorbidities, selecting re-staging time points, adjusting the treatment regime, or choosing the optimal schedule for long-term patient follow-up.

Overall, four main research agenda topics emerge from this comprehensive literature review for clinical image-based AI in PDAC (Table 5). First, there is an urgent need for more and good quality data. Large, well-curated, multi-institutional private and public PDAC datasets are essential for AI development and testing. This allows deep neural networks to extract powerful predictive and diagnostic biomarkers that generalise well in multiple cohorts. Second, easily accessible radiomics AI still dominates the field, with comparatively little work on much more powerful deep learning CNNs. As data availability and quality increases, research should focus on developing models that are entirely and exclusively data-driven. Third, the entire research field needs to globally shift to using better-quality ground truths that represent actual clinical endpoints (such as OS and disease-free survival) as the gold standard for model development. Clinical guideline parameters such as TNM staging, histopathology-based tumour response scores, margin status, and RECIST are hardly predictive of patient outcomes and should not be considered a valid outcome for AI model development. AI in PDAC should improve current clinical workflow rather than replicate/automate existing ineffective practices. Finally, the realm of multimodal AI for PDAC remains unexplored. In a complex and heterogeneous disease such as PDAC, combining information from imaging, histopathology, genetics, and clinical records is crucial to discovering meaningful patterns in the data and building robust prediction models. AI-based imaging biomarkers that stratify PDAC phenotypes predictive of outcome can support individualised care, for instance through the development of pharmacogenomic treatment regimes.

## 6. Conclusions

In conclusion, the future of AI in PDAC lies in addressing the relevant clinical questions, establishing multi-institutional collaborations for the curation of large-scale datasets, and integrating multiple data modalities. By putting forward these issues in the context of current image-based AI literature for PDAC, we hope to help advance meaningful research that will ultimately translate into the improvement of PDAC outcomes, by helping to select the best treatments, for the right patients, at the right time.

## Figures and Tables

**Table 1:** Summary of papers on AI for PDAC detection. The performance for the validation and test sets is reported with respective 95% Confidence Interval or standard deviation when it was provided.

Authors (Year)	Data	Approach	Model	Metric	Validation Performance	Test Performance	Dev. Cohort	Test Cohort
Alves et al. (2022) [35]	CT	DL	3D nnU-Net	AUC	0.991 (0.970–1.0)	** 0.889 (0.833–0.946)	242	** 361
Wang et al. (2021) [38]	CT	DL	2D U-Net	SEN, SPE	0.998, 0.965	..	800	..
Liu et al. (2020) [37]	CT	DL	2D VGG	AUC	1.000 (0.999–1.000)	* 0.997 (0.992–1.000) * 0.999 (0.998–1.000) ** 0.920 (0.891–0.948)	412	* 139 * 189 ** 363
Ma et al. (2020) [39]	CT	DL	2D 4-layer CNN	AUC	0.9652	..	412	..
Tonozuka et al. (2020) [42]	EUS	DL	2D 7-layer CNN	AUC	0.924	* 0.940	93	* 47
Qiu et al. (2021) [43]	CT	Radiomics	SVM	AUC	0.88 (0.84–0.92)	* 0.79 (0.71–0.87)	312	* 93
Chen et al. (2021) [36]	CT	Radiomics	XGBoost	AUC	..	* 0.98 (0.96–0.99) ** 0.76 (0.71–0.82)	944	* 383 ** 212
Chu et al. (2020) [40]	CT	Radiomics	RF	SEN, SPE, ACC	0.950, 0.923, 0.936	..	380	..
Chu et al. (2019) [44]	CT	Radiomics	RF	AUC	..	* 0.999	255	* 125
Li et al. (2018) [41]	^18^FDG PET-CT	Radiomics	SVM-RF	SEN, SPE, ACC	0.952 ± 0.009, 0.975 ± 0.004, 0.965 ± 0.007	..	80	..
Ozkan et al. (2015) [45]	EUS	Radiomics	ANN	SEN, SPE, ACC	..	* 0.833 ± 0.112, 0.933 ± 0.075, 0.875 ± 0.047	172	* 72 images

** external test set, * internal test set. Abbreviations are: DL—deep learning, XGBoost—extreme gradient boost, SVM—support vector machine, VGG—visual geometry group, RF—random forest, ANN—artificial neural network, CNN—convolutional neural network, AUC—area under the receiver operating characteristic curve, SEN—sensitivity, SPE—specificity, ACC—accuracy, Dev. Cohort—development cohort (training + validation).

**Table 2:** Summary of papers on AI for PDAC differential diagnosis. The performance for the validation and test sets is reported with respective 95% Confidence Interval or standard deviation when it was provided.

Authors (Year)	Tissues of Interest	Data	Approach	Model	Metric	Validation Results	Test Results	Dev. Cohort	Test Cohort
Si et al. (2021) [46]	PDAC, IPMN, SCN, other	CT	DL	ResNet + U-Net	ACC	..	** 0.827	319	** 347
Naito et al. (2021) [47]	PDAC	WSI	DL	EfficientNet-B1	AUC	..	* 0.984 (0.960–0.998)	413	* 120
Fu et al. (2021) [8]	PDAC	WSI	DL	Inception + U-Net	ACC	..	* 1.0 ** 0.904	90	* 47 ** 52
Kriegsmann et al. (2021) [48]	PDAC	WSI	DL	EfficientNet	BACC	..	* 0.921	201	..
Ziegelmayer et al. (2020) [50]	PDAC, AIP	CT	DL	RF	AUC	0.90 ± 0.02	..	86	..
Liu et al. (2019) [51]	PDAC	CT	DL	Faster R-CNN	AUC	..	* 0.9632	238	* 100
Saftoiu et al. (2015) [52]	PDAC, MFP	EUS	ML	2-layer ANN	SEN, SPE	..	* 0.946 (0.882–0.978), 0.944 (0.839–0.986)	142	* 25
Ebrahimian et al. (2021) [53]	Benign vs Malignant	CT	Radiomics	RF	AUC	..	* 0.76	59	* 44
Deng et al. (2021) [49]	PDAC, MFP	MR	Radiomics	SVM	AUC	0.997 (0.990–1.0)	** 0.962 (0.907–1.0)	64	** 55
Ma et al. (2021) [54]	PDAC, CP	CT + clinical	Radiomics	LASSO	AUC	0.980 (0.961–1.000)	..	175	..
Liu et al. (2021) [55]	PDAC, AIP	^18^FDG PET-CT	Radiomics	SVM	AUC	0.966 ± 0.008	..	112	..
Ren et al. (2020) [56]	PDAC, PAC	CT	Radiomics	RF	AUC	0.82	..	112	..
Ren et al. (2020) [57]	PDAC, MFP	CT	Radiomics	RF	AUC	0.98 (0.97–1.0)	..	109	..
Park et al. (2020) [58]	PDAC, AIP	CT	Radiomics	RF	AUC	..	* 0.975 (0.936–1.0)	120	* 62
He et al. (2019) [59]	PDAC, PNEN	CT	Radiomics	LASSO	AUC	0.960 (0.942–0.979)	* 0.884 (0.831–0.927)	100	* 47
Ren et al. (2019) [60]	PDAC, MFP	CT	Radiomics	LR	AUC	..	* 0.9	109	* 40
Zhang et al. (2019) [61]	PDAC, AIP	^18^FDG PET-CT	Radiomics	SVM- RF	AUC	0.93	..	111	..
Saftoiu et al. (2012) [62]	PDAC, CP	EUS	Radiomics	2-layer ANN	AUC	0.94 (0.91–0.97)	..	258	..

** external test set, * internal test set. Abbreviations are: MFP—mass-forming pancreatitis, CP—chronic pancreatitis, AIP—autoimmune pancreatitis, IPMN—intraductal papillary mucinous neoplasm, SCN—serous cystic neoplasm, PNEN—pancreatic neuroendocrine neoplasms, PAC—pancreatic adenosquamous carcinoma, DL—deep learning, ML—machine learning, SVM—supported vector machine, RF—random forest, LASSO—least absolute shrinkage and selection operator, LR—logistic regression, ANN—artificial neural network, AUC—area under the receiver operating characteristic curve, SEN—sensitivity, SPE—specificity, BACC—balanced accuracy, Dev. Cohort—development cohort (training + validation).

**Table 3:** Summary of papers on AI for stratification of PDAC patients. The performance for the validation and test sets is reported with respective 95% Confidence Interval or standard deviation when it was provided.

Authors (Year)	Ground Truth	Data	Approach	Model	Metric	Validation Performance	Test Performance	Dev. Cohort	Test Cohort
An et al. (2021) [63]	LNM	CT + clinical	DL	Resnet-18	AUC	0.90 (0.88–0.92)	* 0.92 (0.91–0.92)	113	* 35
Chaddad et al. (2020) [64]	Short term vs. long-term survival	CT	DL + ML	CNN + RF	AUC	0.72	..	159	..
Song et al. (2013) [67]	Grading 1 vs. 2	WSI	ML	SVM	AUC	0.79	..	240	..
Bianet al. (2022) [68]	LNM	MR	Radiomics	LR	AUC	0.75 (0.68–0.82)	* 0.81 (0.69–0.94)	180	* 45
Shi et al. (2022) [65]	LNM	MR + clinical	Radiomics	LR	AUC	0.909 (0.854–0.964)	* 0.835 (0.751–0.919) ** 0.805 (0.720–0.890)	199	** 52
Bian et al. (2021) [69]	TIL	MR	Radiomics	XGBoost	AUC	0.86 (0.79–0.93)	* 0.79 (0.64–0.93)	116	* 40
Cen et al. (2021) [70]	Stage I–II vs. Stage III–IV	CT	Radiomics	LR	AUC	0.940 (0.871–0.979)	* 0.912 (0.781–0.978)	94	* 41
Zhang et al. (2021) [71]	Liver metastasis vs. other metastasis	CT	Radiomics	RF	AUC	0.81	..	77	..
Xing et al. (2021) [72]	Grading 1 vs. 2/3	^18^FDG PET-CT	Radiomics	XGBoost	AUC	..	* 0.921 (0.846–0.996)	99	* 50
Kaissis et al. (2020) [73]	QMS	CT	Radiomics	RF	AUC	0.93 ± 0.01	..	181	..
Chen et al. (2020) [74]	PV-SMV invasion	CT	Radiomics	ElasticNet	AUC	0.871 (0.795–0.946)	* 0.848 (0.724–0.971)	88	58
Liu et al. (2020) [75]	LNM	CT	Radiomics	LR	AUC	0.841 (0.768–0.925)	..	85	..
Li et al. (2020) [76]	LNM	CT + clinical	Radiomics	LR	AUC	..	* 0.912 (0.778–1)	118	*41
Chang et al. (2020) [66]	Grading 1/2 vs. 3	CT	Radiomics	LASSO	AUC	0.961 (0.935–0.987)	* 0.91 (0.864–0.956) ** 0.77 (0.661–0.878)	151	* 150 ** 100
Longlong et al. (2020) [77]	Grading 1 vs. 2 vs. 3	CT	Radiomics	RF	AUC	0.77 (0.64–0.87)	* 0.70 (0.47–0.86)	58	* 25
Qiu et al. (2019) [78]	Grading 1/2 vs. 3	CT	Radiomics	SVM	SEN, SPE, ACC	78 95 86	..	56	..

** external test set, * internal test set. Abbreviations are: LNM—lymph node metastasis, TIL—tumour infiltrating lymphocytes, Grading—grade comparison (low vs. high), QMS—quasi mesenchymal subtype, PV-SMV—portal vein superior mesenteric vein, DL—deep learning, ML—machine learning, SVM—supported vector machine, RF—random forest, LR—logistic regression, CNN—convolutional neural network, XGBoost—extreme gradient boost, AUC—area under the receiver operating characteristic curve, SEN—sensitivity, SPE—specificity, ACC—accuracy, Dev. Cohort—development cohort (training + validation).

**Table 4:** Summary of papers on AI for PDAC treatment response prediction. The performance for the validation and test sets is reported with respective 95% Confidence Interval or standard deviation when it was provided.

Authors (Year)	Treatment	Predict	Data	Approach	Model	Metric	Validation Results	Test Results	Dev. Cohort	Test Cohort
Yao et al. (2021) [81]	Resection	OS	CT	DL	Conv- LSTM	CI	0.667	..	296	..
Zhang et al. (2020) [80]	Resection	OS	CT	DL	CNN	CI	..	** 0.651	68	** 30
Watson et al. (2020) [82]	Chemotherapy	PR vs. NR	CT + clinical	DL	LeNet	AUC	..	* 0.785	..	* 65
Zhang et al. (2021) [83]	Resection	2-year survival	CT	DL + ML	RF	AUC	..	* 0.84 (0.70–0.98)	68	* 30
Li et al. (2021) [84]	Resection	1-year and 2-year recurrence risk	CT + clinical	DL + ML	ANN	AUC	0.916 (0.860–0.955) 0.872 (0.809–0.921)	** 0.764 (0.644–0.859) ** 0.773 (0.654–0.866)	153	** 47
Zhang et al. (2020) [83]	Resection	Death risk	CT	DL + ML	RF	AUC	0.72 (0.58–0.86)	** 0.81 (0.64–0.98)	68	** 30
Healy et al. (2021) [79]	Resection	OS	CT	Radiomics	CPH	CI	0.626 (0.625–0.627)	** 0.545 (0.543–0.546)	352	** 215
Shi et al. (2021) [85]	Resection	OS	CT	Radiomics	CPH	CI	0.74 (0.70–0.78)	* 0.73 (0.66–0.79)	210	* 89
Wei et al. (2021) [86]	Resection	1-year RFS	^18^FDG PET-CT	Radiomics	CPH	CI	0.890 (0.835–0.945)	* 0.865 (0.778–0.952)	109	* 47
Xie et al. (2020) [87]	Resection	OS	CT	Radiomics	CPH	CI	..	* 0.726 (0.646–0.806)	147	* 73
Park et al. (2020) [88]	Resection	OS	CT	Radiomics	RF	CI	0.74	..	153	..
Parr et al. (2020) [89]	Radiotherapy	OS	CT	Radiomics	CPH	CI	0.68	..	74	..
Kaissis et al. (2020) [90]	Resection	OS	CT + clinical + genomics	Radiomics	LPCA	CI	0.65 (0.60–0.69)	..	103	
Hui et al. (2020) [91]	Resection margin	R0 vs. R1	CT	Radiomics	SVM	AUC	0.8641	..	86	..
Bian et al. (2020) [92]	Resection margin	R0 vs. R1	CT	Radiomics	LR	AUC	0.750 (0.672–0.824)	..	181	..
Tang et al. (2019) [93]	Resection	NER (>12 months) vs. ER (<12 months)	MR	Radiomics	LR	AUC	0.802 (0.721–0.868)	* 0.807 (0.677–0.902) ** 0.781 (0.699–0.850)	177	* 74 ** 126
Zhou et al. (2019) [94]	Irradiation stent	RSFS	CT	Radiomics	CPH	CI	0.791 (0.614–0.967)	* 0.779 (0.504–1.000)	74	* 32
Cozzi et al. (2019) [95]	Radiotherapy	OS	CT	Radiomics	CPH	CI	..	* 0.75 ± 0.03	60	* 40
Kaissis et al. (2019) [96]	Chemotherapy	OS	MR	Radiomics	GBDT	CI	0.71 (0.60–0.80)	..	55	..
Kaissis et al. (2019) [97]	Resection	Above vs. below average OS	MR	Radiomics	RF	AUC	0.93 ± 0.07	* 0.9	102	* 30
Chakraborty et al. (2017) [98]	Resection	Survival < 2 years vs. survival > 2 years	CT	Radiomics	Bayes	AUC	0.9	..	35	..
Cui et al. (2016) [99]	Radiotherapy	OS	^18^FDG PET-CT	Radiomics	CPH	CI	0.623	* 0.661 (0.418–0.841)	90	* 49

** external test set, * internal test set. Abbreviations are: PR—pathological response, NR—no response, NER—non-early recurrence, ER—early recurrence, RFS—recurrence-free survival, RSFS—restenosis-free survival, DL—deep learning, ML—machine learning, SVM—supported vector machine, RF—random forest, CPH—Cox proportional hazard, GBDT—gradient-boosted decision tree, LPCA—linear principle component analysis, LR—logistic regression, ANN—artificial neural network, AUC—area under the receiver operating characteristic curve, CI—concordance index, Dev. Cohort—development cohort (training + validation).

**Table 5:** Overview of the main topics for future clinical AI research in PDAC imaging.

Research Agenda for Clinical AI in PDAC Imaging
- To acquire more, good quality data coming from large, well-curated, multi-institutional private and public PDAC datasets - To switch focus towards state-of-the-art, entirely data-driven deep learning models - To use better quality ground truths that represent actual clinical endpoints such as overall survival and disease-free survival as the gold standard for model development - To investigate the use of multimodal AI, combining information from imaging, histopathology, genetics and clinical records

## Appendix A

This scoping review adhered to the PRISMA guidelines. Searches were conducted on PubMed, Web of Science, Cochrane, and Embase on 14 September 2021, and updated on 25 January 2022. The used search string was the following.

**Table A1:** Search strategy. MeSH terms and keywords for the included databases.

Database	Search Strategy
**PubMed**	(“Pancreatic Neoplasms”(Mesh:NoExp) OR “Carcinoma, Pancreatic Ductal”(Mesh) OR “Pancreatic Intraductal Neoplasms”(Mesh) OR (Pancrea*(tiab) AND (Neoplasm*[tiab] OR cancer*[tiab] OR Carcinoma*[tiab] OR Adenocarcinoma*[tiab])) OR PDAC(tiab)) AND (“Artificial Intelligence”(Mesh) OR AI(tiab) OR Artificial Intelligence(tiab) OR CNN(tiab) OR Convnet(tiab) OR Deep Learning(tiab) OR Machine learning (tiab) OR Neural network*(tiab) OR pathomic*(tiab) OR radiomic*(tiab) OR supervised Learning(tiab) OR Transfer Learning(tiab) OR Unet(tiab) OR unsupervised Learning(tiab))
**Embase**	(“Pancreatic Neoplasms” or “Carcinoma, Pancreatic Ductal” or “Pancreatic Intraductal Neoplasms” or (Pancrea* and (Neoplasm* or cancer* or Carcinoma* or Adenocarcinoma*)) or PDAC).mp. and (“Artificial IntelligenceOR AI” or CNN or Convnet or “Deep Learning” or “Machine learning” or “Neural network*” or pathomic* or radiomic* or “supervised Learning” or “Transfer Learning” or Unet or “unsupervised Learning”).
**Web of Science**	(TS = ((“Pancreatic Neoplasms” OR “Carcinoma, Pancreatic Ductal” OR “Pancreatic Intraductal Neoplasms” OR (Pancrea* AND (Neoplasm* OR cancer* OR Carcinoma* OR Adenocarcinoma*)) OR PDAC))) AND TS = ((“Artificial Intelligence”OR AI OR CNN OR Convnet OR “Deep Learning” OR “Machine learning” OR “Neural network*” OR pathomic* OR radiomic* OR “supervised Learning” OR “Transfer Learning” OR Unet OR “unsupervised Learning”))
**Cochrane**	(“Pancreatic Neoplasms”(Mesh:NoExp) OR “Carcinoma, Pancreatic Ductal”(Mesh) OR (Pancrea*[tiab] AND (Neoplasm*[tiab] OR cancer*[tiab] OR Carcinoma*[tiab] OR Adenocarcinoma*[tiab])) OR PDA*(tiab)) AND (“Artificial Intelligence”(Mesh) OR AI(tiab) OR Artificial Intelligence(tiab) OR CNN(tiab) OR Convnet(tiab) OR Deep Learning(tiab) OR Machine learning (tiab) OR Neural network*(tiab) OR pathomic*(tiab) OR radiomic*(tiab) OR supervised Learning(tiab) OR Transfer Learning(tiab) OR Unet(tiab) OR unsupervised Learning(tiab))

Two independent reviewers (M.S. and N.A.) screened the titles and subsequently reviewed all full-text articles based on preselected variables to compare results. A third independent expert reviewer (P.V.) reviewed the full-text articles for digital pathology imaging. Conflicting evaluations were resolved by discussions between the three reviewers.

Inclusion criteria and corresponding number of records excluded at each step (n) were the following:

Full-read criteria:

(1) Study was published in a peer-reviewed journal and was not an abstract, review paper, conference paper, commentary, editorial, or not available. (n = 293)
(2) Study considered patients clinically diagnosed with pancreatic cancer (pancreatic ductal adenocarcinoma or non-specified type of pancreatic cancer). (n = 343)
(3) Study used AI to predict an outcome related to one of the following tasks (n = 289):
  ○ Detection: determine the presence or absence of PDAC in an input image with or without localization of the tumours.
  ○ Diagnosis: differentiate between PDAC and other benign and/or malignant pancreatic lesions.
  ○ Staging: stratify PDAC patients into different subgroups based on clinical/histopathological outcomes or survival.
  ○ Treatment indication: predict treatment response based on pre-treatment data.
  ○ Treatment response prediction: monitor treatment response based on follow-up data.
(4) Study used medical images as input. Included modalities were computed tomography (CT), magnetic resonance (MR), ultrasound (EUS), 18fluoro-2-deoxy-d-glucose positron emission tomography/computed tomography (18FDG PET/CT), and whole-slide images (WSI). (n = 56)

Qualitative synthesis criteria:

(5) Study included a population larger than 20 patients. (n = 3)
(6) Clinical information regarding patients in the used cohorts was available. Minimum required fields were: number of patients, age distribution, and sex distribution. (n = 14)
(7) Study reports results in at least one patient cohort not used to train the model. (n = 3)
(8) Study reports how the results were obtained. (n = 6)

A data selection template was designed to evaluate research characteristics and performance. All variables are evaluated independently by the three reviewers (M.S., N.A., and P.V.).

The template included the following fields:

- Modality (CT, MR, EUS, 8FDG PET/CT, WSI)
- Task (detection, diagnosis, staging, treatment, monitoring)
- Tissue of interest (PDAC, pancreatic cancer)
- Tumour location (head, neck, body, tail)
- Disease stage (I, II, III, IV)
- Therapy
- Type of AI (Radiomics, Deep Learning, other)
- Model
- Ground truth
- Performance metric
- Type of internal validation (single split, cross-validation)
- Performance on training set
- Performance on internal validation set
- Performance on internal test set
- Performance on external test set
- Cohort size for development set (train/validation)
- Patient distribution for development set with regard to considered ground truth
- Cohort size for internal test set
- Patient distribution for internal test set with regard to considered ground truth
- Cohort size for external test set
- Patient distribution for external test set with regard to considered ground truth
